# Perceived artificial intelligence readiness in medical and health sciences education: a survey study of students in Saudi Arabia

**DOI:** 10.1186/s12909-025-06995-1

**Published:** 2025-03-26

**Authors:** Manal Almalki, Moh A. Alkhamis, Farah M. Khairallah, Mohamed-Amine Choukou

**Affiliations:** 1 Department of Public Health, College of Nursing and Health Sciences, Jazan University, Jazan, 45142 Saudi Arabia; 2 Department of Epidemiology and Biostatistics, Faculty of Public Health, Health Sciences Centre, Kuwait University, Kuwait City, Kuwait; 3 Department of Occupational Therapy, College of Rehabilitation Sciences, Rady Faculty of Health Sciences, University of Manitoba, Winnipeg, Manitoba Canada

**Keywords:** Artificial intelligence, Medical education, AI readiness, Ethics, Medical students, Curriculum reform, Saudi Arabia

## Abstract

**Background:**

As artificial intelligence (AI) becomes increasingly integral to healthcare, preparing medical and health sciences students to engage with AI technologies is critical.

**Objectives:**

This study investigates the perceived AI readiness of medical and health sciences students in Saudi Arabia, focusing on four domains: cognition, ability, vision, and ethical perspectives, using the Medical Artificial Intelligences Readiness Scale for Medical Students (MAIRS-MS).

**Methods:**

A cross-sectional survey was conducted between October and November 2023, targeting students from various universities and medical schools in Saudi Arabia. A total of 1,221 students e-consented to participate. Data were collected via a 20-minute Google Form survey, incorporating a 22-item MAIRS-MS scale. Descriptive and multivariate statistical analyses were performed using Stata version 16.0. Cronbach alpha was calculated to ensure reliability, and least squares linear regression was used to explore relationships between students’ demographics and their AI readiness scores.

**Results:**

The overall mean AI readiness score was 62 out of 110, indicating a moderate level of readiness. Domain-specific scores revealed generally consistent levels of readiness: cognition (58%, 23.2/40), ability (57%, 22.8/40), vision (54%, 8.1/15) and ethics (57%, 8.5/15). Nearly 44.5% of students believed AI-related courses should be mandatory whereas only 41% reported having such a required course in their program.

**Conclusions:**

Medical and health sciences students in Saudi Arabia demonstrate moderate AI readiness across cognition, ability, vision, and ethics, indicating both a solid foundation and areas for growth. Enhancing AI curricula and emphasizing practical, ethical, and forward-thinking skills can better equip future healthcare professionals for an AI-driven future.

**Supplementary Information:**

The online version contains supplementary material available at 10.1186/s12909-025-06995-1.

## Introduction

How prepared are students to embrace AI in medical and health sciences education and future practice? AI-powered health technologies are expected to become increasingly integrated into patient care globally, enhancing access, driving digital transformation, and supporting evidence-based practices [1, 2]. However, standardized clinical adoption of AI across all disciplines remains limited [3, 4]. So far, successful AI implementations have mainly focused on operational support and diagnostic applications, such as reading imaging scans, endoscopy, and pathology reports (e.g [5–7]). Ultimately, embracing future changes in healthcare hinges on education [8]. Understanding perceptions of AI in healthcare is crucial for successful deployment and adoption, particularly among medical and health sciences students who will soon become healthcare leaders and providers of essential services [9–11].

Drawing on Bloom’s Taxonomy of Learning Domains [9, 12] educational readiness must involve three components: cognitive (knowledge), affective (attitudes), and psychomotor (practical skills). A growing body of literature highlights the need to cultivate AI awareness and knowledge in healthcare [13–16] to better prepare health sciences students to work with AI technologies [17]. Incorporating AI content into health sciences curricula can help students acquire the necessary AI related skills [18, 19]. Previous studies show that many students demonstrate some awareness of AI in healthcare (e.g [19, 20]), reinforcing the call for broader incorporation of AI topics and applications in medical and health sciences education.

To improve the current curricula, large-scale surveys should be conducted to assess the attitudes and perceptions of medical and health sciences students regarding AI content [1]. These assessments can identify competencies for students, as well as the resources and knowledge that faculty need to prioritize [1]. Although such research is highly needed, the literature on the topic is rather rare [17, 19, 21, 22]. Moreover, disparities exist among different medical and health sciences disciplines, with relatively little literature on AI applications in fields like rehabilitation, public health, pharmacy, and nursing [21, 23] compared to medicine and dentistry [10, 11, 24, 25]. Consequently, a balanced approach is needed to identify the educational needs, preferences, and capacities of students across different disciplines. For example, a recent scoping review [23] on the impact of AI on nursing education highlights the need for curricular reform in academic and clinical context to prepare nurses and nursing students for safe and efficient practice in the AI era. Similar strategies should focus on enhancing awareness, knowledge, and skills related to AI in healthcare, while aligning these efforts with faculty expertise and available technological and pedagogical resources [3].

A recent study conducted in Saudi Arabia [9] revealed positive attitudes among medical students toward AI education, yet its focus was limited to a single discipline, underscoring the need for interdisciplinary research. Using Bloom’s Taxonomy as a guiding framework, this study explores the perceived AI readiness of medical and health sciences students in Saudi Arabia. We address two key Research Questions (RQ): (1) What are the overall levels of AI readiness among medical and health sciences students in Saudi Arabia? And (2) Which demographic and educational factors (e.g., year of study, program type, perceived adequacy of AI-related training, and attitudes toward AI course requirements) are associated with variations in these AI readiness domains? The findings will inform curriculum development and enhance interprofessional collaboration in the era of AI-driven healthcare.

## Methods

### Study design and population

A cross-sectional study was conducted between October and November 2023. Participants were recruited using a convenience sampling via social media platforms (i.e., WhatsApp, Snapchat, X (formerly Twitter), and Telegram). These platforms were selected due to their popularity among Saudi university students [26, 27]. Eligible participants included undergraduate and post graduate students aged 18 years or older, currently enrolled in universities and medical schools in Saudi Arabia, and capable of providing informed consent. A built-in electronic consent form was completed by each participant at the beginning of the survey. Ethical approval for the study was obtained from the Standing Committee for Publication and Research Ethics at Jazan University (Reference No. REC-45/04/820, obtained October 24, 2023). The study adhered to the Declaration of Helsinki. The questionnaire was pretested with eight university students to refine clarity and structure.

### Data collection

Data were collected via a 20-minute Google Form questionnaire, which included three sections: demographics (e.g., age, gender, academic program type); students’ perceptions of AI-related course requirements; and AI-related questions from the Medical Artificial Intelligences Readiness Scale for Medical Students (MAIRS-MS) [28]. The MAIRS-MS, a validated 22-item instrument, was selected to assess students’ cognition, ability, vision, and ethics regarding AI readiness [29]. The survey was distributed in English only, as English is the medium of instruction for medical and health sciences education in Saudi Arabia.

### Statistical analysis

We used Stata version 16.0 to infer all of our statistical analyses [30]. No missing data were present as all questions were mandatory; however, incomplete surveys were excluded because participants voluntarily withdrew or discontinued their participation before completing the questionnaire. The outcome variables included cognition, ability, vision, and ethics scores extracted from MAIRS-MS 5-point Likert subscales, where 1 indicated strong disagreement and 5 indicated strong agreement, with higher scores indicating greater readiness. Internal consistency for each outcome was evaluated by calculating the Cronbach alpha reliability coefficients (α). Estimated α values between 0.6 and 0.8 for the general scale and the subscale are deemed acceptable [31].

Predictor variables comprised participants’ demographics and their general attitudes toward AI. Frequencies and relative frequencies were used to summarize these variables. We assessed the normality of the outcome variable using the Shapiro-Wilk (*S-W*) test. We found that our outcome variables were not normally distributed (*p*-values < 0.05), and therefore, we explored several statistical transformations (i.e., subtracting individual scores from the mean score and dividing it by the standard deviation) to achieve normal distribution (*S-Wp*-values > 0.1). The mean scores represented the midpoint of participants’ AI readiness rather than a definitive level of preparedness, as psychometric scales are designed to approximate normal distributions. Floor and ceiling effects were assessed by calculating the proportion of participants scoring at the minimum and maximum levels, with a threshold of 15% used to determine significant effects. The results of this analysis indicated that floor and ceiling effects were minimal and did not impact the interpretation of the findings.

Exploratory Factor Analysis was performed to evaluate the factor structure of the MAIRS-MS questionnaire. We used least squares linear regression for each outcome to model multivariate relationships with the selected predictors. We assessed the statistical significance of all possible two-way interactions between predictors to explore any plausible interactions between our variables, bearing in mind our study is exploratory in nature. We visually inspected residual vs. fitted values plots to ensure that our final models do not violate the assumptions of the linear regression. The Variance Inflation Factor (VIF) approach was employed to ensure no collinearity issues.

## Results

### Participants’ demographic characteristics

A total of 1221 participants completed our questionnaire. Table 1 summarizes the participants’ baseline characteristics and general attitudes toward AI. Most participants were females (67.0%) and between 18 and 22 years old (49.7%). Approximately 70% of the students were enrolled in bachelor’s degree, diploma, or higher diploma or equivalent in medical and clinical-related sciences. The majority of participating were clustered evenly between year 1 and 4 of their programs.

We found that 44.5% of the participants believed that AI-related courses must be required, while 41% reported having a required AI-related course in their curriculum. However, 40% of the students totally disagreed that the knowledge and training provided by these courses were adequate for applying AI in healthcare (Table 1).

**Table 1:** Demographic characteristics of the participants (*N* = 1221)

Variable	*n* (%)
**Age group**	
18–22	607 (49.7)
23–34	404 (33.1)
35–44	83 (6.8)
45–54	61 (5.0)
55–64	23 (1.9)
65–74	19 (1.6)
75 years or older	24 (2.0)
**Gender**	
Female	818 (67.0)
Male	281 (23.0)
Prefer not to say	122 (10.0)
**What degree are pursuing or currently have?**	
Bachelor’s degree	376 (30.8)
Diploma (after high school) or the equivalent	240 (19.7)
Higher Diploma or the equivalent	230 (18.9)
Master’s degree or the equivalent	195 (16.0)
PhD degree or the equivalent	87 (7.1)
Postdoc	93 (7.6)
**Program**	
Medical & clinical related sciences	898 (73.6)
Health-related sciences	290 (23.8)
Other	33 (2.7)
**Year Level**	
1st	326 (26.7)
2nd	187 (15.3)
3rd	224 (18.4)
4th	207 (17.0)
5th	112 (9.2)
6th	65 (5.3)
7th	100 (9.0)
**Do you believe that Al-related courses must be required or optional in your study program?**	
Required	543 (44.5)
Optional	678 (55.5)
**Do you have any Al-related courses in your study program?**	
Yes, it is a required course	501 (41.0)
Yes, it is an optional course	424 (34.7)
No, we do not have any AI-related courses	296 (24.2)
**Do you believe that the knowledge and training provided by these courses on Al applications in healthcare is adequate?**	
Totally disagree	371 (40.1)
Disagree	200 (21.6)
Neutral	198 (21.4)
Agree	86 (9.3)
Totally agree	70 (7.6)

**Fig. 1:**
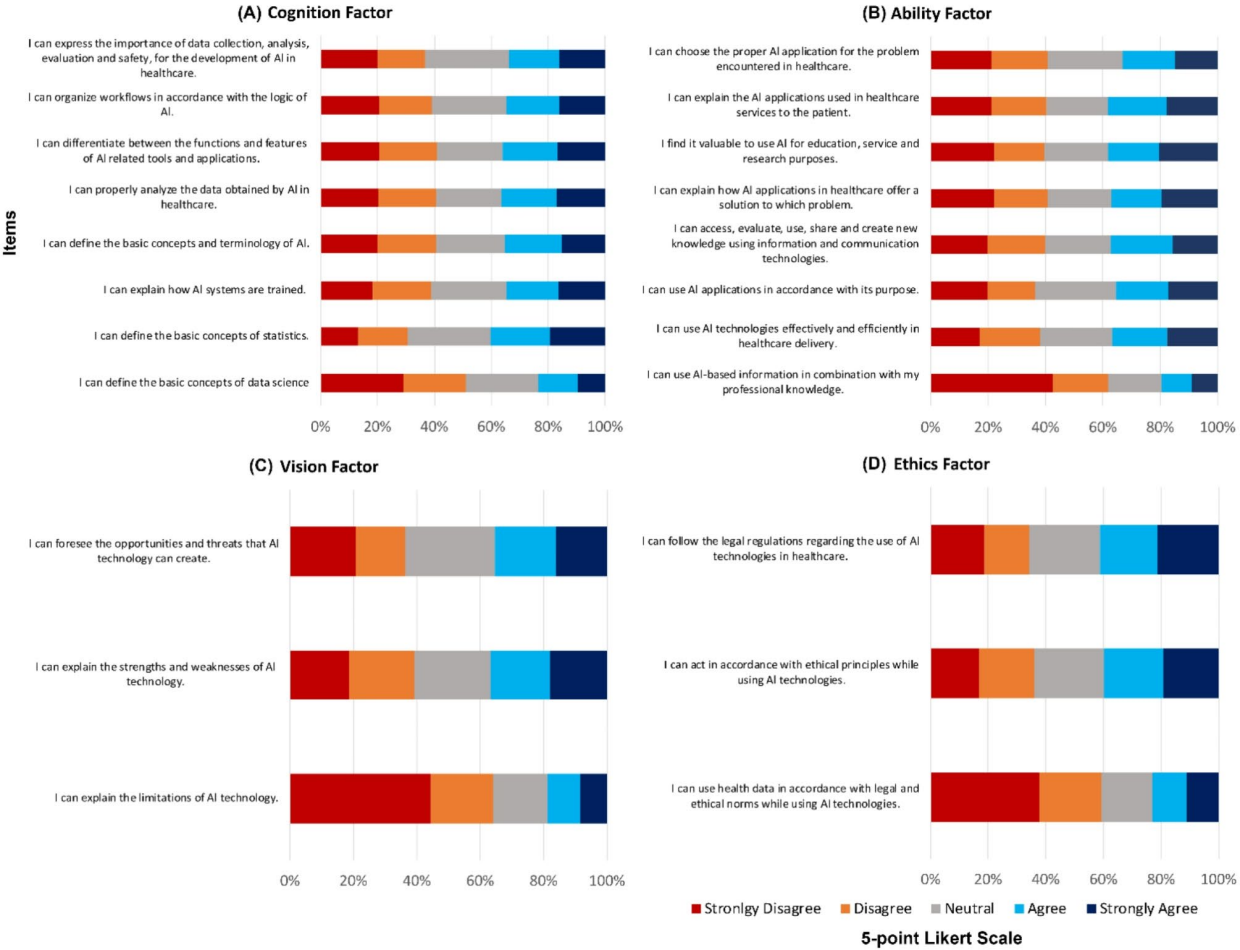
Bar charts summarizing participants’ responses on the Medical Artificial Intelligences and Readiness Scale for Medical Students score for each domain. (**A**) represents cognition. (**B**) Ability. (**C**) Vision. (**D**) Ethics

### Participants’ AI readiness levels

Figure 1 illustrates the summary results of the participants’ self-rated AI readiness based on MAIRS-MS four domains. Our estimated inter-reliability coefficients for the overall scale and cognition and ability subscales were acceptable (αs > 0.7; Table 2). However, the estimated αs for the vision and ethics subscales were on the borderline of acceptability (αs > 0.5; Table 2). We found that the mean scores for cognition and ability domains were 23.2 and 22.8 out of a possible total of 40, respectively (Table 2). Meanwhile, the mean scores for vision and ethics factors were 8.1 and 8.5 out of 15, respectively. Yet, the overall mean readiness score based on the four domains was 62 out of a possible total of 110.

**Table 2:** Summary statistics of domain scores and their Cronbach alpha reliability coefficients (α)

Factor	Mean score $\:\pm\:$ S.D (possible total score)	$\:\varvec{\alpha\:}$	
Cognition	23.2 $\:\pm\:$ 5.8 (40)	0.71	
Ability	22.8 $\:\pm\:$ 6.3 (40)	0.73	
Vision	8.1 $\:\pm\:$ 2.8 (15)	0.52	
Ethics	8.5 $\:\pm\:$ 2.8 (15)	0.56	
Overall	62 $\:\pm\:$ 8.7 (110)	0.88	

### Demographic and educational predictors of AI readiness

While the inferred R^2^s values were relatively low (Table 3), each domain’s multivariate regression model was overall strongly significant (*p*-values < 0.001). The highest R^2^ was approximately 0.21 for the cognition model, while the lowest R^2^ was 0.09 for the vision model. No statistically significant interactions between predictors were found, and all VIFs were less than 1.2, indicating no collinearity. Year level, the belief that AI-related courses must be required, and the perception that current AI training is inadequate were consistently significant predictors for all four domains (*p*-values < 0.01; Table 3). However, the type of study program was only a significant predictor of both ability and ethics (*p*-values < 0.05; Table 3). Neither age, gender, nor degree type were significant predictors of the four domains.

**Table 3:** Multivariate linear regression models for the readiness domains. Significant regression coefficients, 95% CI, and their *p*-values are boldfaced

**Variable**	Cognition (*R*^2^ = 0.21)		Ability (*R*^2^ = 0.11)		Vision (*R*^2^ = 0.09)		Ethics (*R*^2^ = 0.13)	
	$\:\beta\:$(95% CI*)	*p-value*	$\:\beta\:$(95% CI)	*p-value*	$\:\beta\:$(95% CI)	*p-value*	$\:\beta\:$(95% CI)	*p-value*
Age	0.02 (-0.27, 0.31)	0.90	-0.01 (-0.15, 0.13)	0.88	0.04 (-0.26, 0.36)	0.77	0.04 (-0.10, 0.18)	0.57
Gender	-0.08 (-0.65, 0.48)	0.76	0.05 (-0.21, 0.32)	0.69	-0.03 (-0.63, 0.57)	0.91	-0.21 (-0.49, 0.05)	0.12
Degree	0.07 (-0.16, 0.31)	0.53	0.01 (-0.10, 0.12)	0.91	-0.09 (-0.34, 0.16)	0.48	-0.03 (-0.15, 0.08)	0.57
Program	0.60 (-0.15, 1.30)	0.11	**0.59 (0.22**,** 0.96)**	**< 0.01**	0.57 (-0.24, 1.39)	0.17	**0.43 (0.43**,** 0.05)**	**0.02**
Year level	**0.30 (0.09**,** 0.51)**	**< 0.01**	**0.16 (0.06**,** 0.26)**	**< 0.01**	**0.38 (0.16**,** 0.60)**	**< 0.01**	**0.11 (0.01**,** 0.20)**	**0.04**
AI required or optional	**1.62 (0.86**,** 2.38)**	**< 0.01**	**0.46 (0.1**,** 0.82)**	**0.01**	**0.86 (0.05**,** 1.68)**	**0.03**	**0.50 (0.13**,** 0.87)**	**< 0.01**
Have AI-related courses	0.62 (-0.13, 1.38)	0.10	0.01 (-0.36, 0.37)	0.97	0.55 (-0.25, 1.36)	0.18	0.26 (-0.10, 0.64)	0.15
AI applications adequate	**0.79 (0.49**,** 1.08)**	**< 0.01**	**0.37 (0.23**,** 0.051)**	**< 0.01**	**1.05 (0.74**,** 1.37)**	**< 0.01**	**0.38 (0.23**,** 0.52)**	**< 0.01**

*95% Confidence Interval

## Discussion

Among the convenience sample of students surveyed, medical and health sciences students in Saudi Arabia perceived themselves to be moderately prepared for AI. The overall mean AI readiness score of 62 out of 110 suggests that, while students possess a foundational understanding of AI, there is ample room for growth in knowledge and application of AI in healthcare. Using Bloom’s Taxonomy of Learning Domains as a conceptual framework, we discuss these results in terms of enhancing knowledge (cognitive domain), attitudes and values (affective domain), and practical skills (psychomotor domain), as follows.

### Cognitive domain: reforming curriculum for longitudinal building knowledge

Students’ moderate scores in cognition and ability imply that they have a basic understanding of AI concepts and some confidence in their technical skills. However, the finding that year level was a significant predictor of AI readiness underscores the need for introducing AI-related content early and reinforcing it throughout the educational program. As students progress, increasing depth and complexity in AI topics– ranging from foundational algorithms to clinical decision-support systems– can ensure that their knowledge keeps pace with rapidly evolving technologies [8, 32]. In addition, the perception that current training is inadequate suggests that existing curricula may need to be revamped [3, 33]. Incorporating structured, mandatory AI courses that cover core principles and emerging applications could help bridge the gap [1], raising both the floor and ceiling of students’ cognitive preparedness.

### Affective domain: fostering engaged attitudes and ethical reasoning

Students demonstrated moderate readiness in the vision and ethics domains, with the lowest scores in vision (54%), indicating that they may not fully appreciate the broader implications of AI [34]. The significant relationship between the belief that AI-related courses should be mandatory and improved readiness scores across all domains suggests that students who value structured AI education may also be more inclined to engage ethically and envision AI’s long-term implications. Furthermore, the type of study program was a significant predictor of ability and ethics, indicating that students in certain fields may be more receptive or better prepared to handle AI’s moral and professional challenges. By incorporating ethics case studies, debates on patient privacy and algorithmic bias, and discussions on AI’s future role in patient care, educators can nurture a generation of professionals who are not only knowledgeable but also ethically conscious and forward-thinking [35, 36].

To strengthen the affective domain, educators should incorporate ethics and vision into the curriculum beyond a single lecture or course. Case-based discussions, simulations, and interprofessional workshops can help students explore the complex ethical dilemmas associated with AI [35, 37]. By engaging students in conversations about data privacy, algorithmic bias, AI hallucinations, and long-term implications for patient care, educators can foster more nuanced attitudes that transcend rote memorization and encourage critical reflection [36].

### Psychomotor domain: bridging the gap between theory and practice

Although the cognition and ability scores were moderate, the perception that AI training is inadequate points to gaps in translating theoretical knowledge into hands-on skills [32]. For students to apply their understanding effectively in clinical settings, curricula must include practical exercises, simulations, and opportunities for interprofessional collaboration [3, 33]. Encouraging students to engage with AI tools, analyze clinical scenarios where AI can optimize patient outcomes, and collaboratively work on AI-related projects can advance them through Bloom’s psychomotor domain—from merely knowing what AI is to effectively using it in real-world contexts.

### Strengths and limitations

This study is the first to assess AI readiness among a large sample of medical and health sciences students in Saudi Arabia. Using the validated MAIRS-MS instrument enhances the reliability of our findings. We collected data using convenience sampling, a non-probability technique that may introduce selection bias. However, this approach provided us with a unique opportunity to gather data from multiple universities within a reasonable timeframe. Also, the cross-sectional design limits the ability to establish causality between variables and outcomes in this study [38]. Also, reliance on self-reported data may introduce bias due to inaccurate reporting [39], and the English version of the MAIRS-MS scale may limit generalizability of our results [29]. We acknowledge that the alphas for the vision and ethics subscales were on the borderline of acceptability, and we recognize that this may downplay concerns about internal consistency. In the current study, we did not explore alternative factor structures, but we encourage future research to consider this approach to improve scale reliability. It is important to interpret our findings within the context of these statistical limitations. We also advocate for further validation of the MAIRS-MS scale or the development of more robust scales to address these issues in future work. Furthermore, to address these limitations, future research should validate the MAIRS-MS in diverse linguistic settings and Arab cultural contexts. Additionally, incorporating qualitative methods [3] can offer deeper insights, exploring how instructors’ attitudes and students’ perceptions of AI’s influence on healthcare roles shape their readiness. Such investigations will provide a more holistic understanding of how to best prepare medical and health sciences students for an AI-driven future.

## Conclusions

Medical and health sciences students in this study demonstrated a generally moderate level of AI readiness across cognition, ability, vision, and ethics, indicating a solid starting point yet substantial room for improvement. By systematically enhancing foundational knowledge (cognitive domain), cultivating engaged and forward-looking attitudes (affective domain), and providing hands-on, practical experiences (psychomotor domain), educators can better prepare future healthcare professionals to integrate AI ethically and effectively into patient care. Future research should investigate the relative impact of different educational strategies and compare AI training and preparedness across diverse regions and countries. Such comparative efforts can guide the development of standardized, evidence-based guidelines for integrating AI into medical and health sciences curricula, ensuring that graduates are both technically proficient and ethically grounded in harnessing AI’s transformative potential.

## Electronic supplementary material

Below is the link to the electronic supplementary material.

Supplementary Material 1

## Data Availability

All data generated or analyzed during this study are included in this published article. The datasets used and/or analyzed during the current study are available from the corresponding author on reasonable request.

## Abbreviations

AI: Artificial intelligence
MAIRS-MS: Medical artificial intelligences readiness scale for medical students
