# Supplementary material for: Perceived artificial intelligence readiness in medical and health sciences education: a survey study of students in Saudi Arabia
Source: BMC Med Educ. 2025 Mar 26;25:439. doi: 10.1186/s12909-025-06995-1 (PMC11938701; doi:10.1186/s12909-025-06995-1)
Supplement: Supplementary file 1 — Supplementary Material 1 [file 12909_2025_6995_MOESM1_ESM.docx]

| Section 1: Tell us about your self |
| --- |
| Age |
| 18-22 |
| 23-34 |
| 35-44 |
| 45-54 |
| 55-64 |
| 65-74 |
| 75 years or older |
| Gender |
| Female |
| Male |
| Prefer not to say |
| What degree are pursuing or currently have? |
| Bachelor's degree |
| Diploma (after high school) or the equivalent |
| Higher Diploma or the equivalent |
| Master’s degree or the equivalent |
| PhD degree or the equivalent |
| Postdoc |
| Program |
| Medical & clinical related sciences |
| Health related sciences |
| Other |
| Year level |
| 1st |
| 2nd |
| 3rd |
| 4th |
| 5th |
| 6th |
| 7th |

| Section 2: Your perceptions of AI-related course requirements |
| --- |
| Do you believe that Al-related courses need to be required or optional in your study program? |
| Required |
| Optional |
| Do you have any Al-related courses in your study program? |
| Yes, it is a required course |
| Yes, it is an optional course |
| No, we do not have any AI-related courses |
| Do you believe that the knowledge and training provided by these courses on Al applications in healthcare is adequate? |
| Totally disagree |
| Disagree |
| Neutral |
| Agree |
| Totally agree |

**Section 3: Medical Artificial Intelligence Readiness Scale for Medical Students (MAIRS-MS)**

| 1-Strongly Disagree | 2-Disagree | 3-Neutral | 4-Agree | 5-Strongly Agree |
| --- | --- | --- | --- | --- |

| 1. I can define the basic concepts of data science |
| --- |
| 1. I can define the basic concepts of statistics |
| 1. I can explain how AI systems are trained |
| 1. I can define the basic concepts and terminology of AI |
| 1. I can properly analyze the data obtained by AI in healthcare. |
| 1. I can differentiate the functions and features of AI related tools and applications. |
| 1. I can organize workflows compatible with AI. |
| 1. I can express the importance of data collection, analysis, evaluation and safety; for the development of AI in healthcare. |
| 1. I can harness AI-based information combined with my professional knowledge. |
| 1. I can use AI technologies effectively and efficiently in healthcare delivery. |
| 1. I can use artificial intelligence applications in accordance with its purpose. |
| 1. I can access, evaluate, use, share and create new knowledge using information and communication technologies. |
| 1. I can explain how AI applications offer a solution to which problem in healthcare. |
| 1. I find valuable to use AI for education, service and research purposes. |
| 1. I can explain the AI applications used in healthcare services to the patient. |
| 1. I can choose proper AI application for the problem encountered in healthcare. |
| 1. I can explain the limitations of AI technology. |
| 1. I can explain the strengths and weaknesses of AI technology. |
| 1. I can foresee the opportunities and threats that AI technology can create. |
| 1. I can use health data in accordance with legal and ethical norms. |
| 1. I can conduct under ethical principles while using AI technologies. |
| 1. I can follow legal regulations regarding the use of AI technologies in healthcare. |
